# Effect of Doxycycline on Survival in Abdominal Aortic Aneurysms in a Mouse Model

**DOI:** 10.1155/2021/9999847

**Published:** 2021-04-27

**Authors:** Lisa C. Adams, Julia Brangsch, Jan O. Kaufmann, Dilyana B. Mangarova, Jana Moeckel, Avan Kader, Rebecca Buchholz, Uwe Karst, Rene M. Botnar, Bernd Hamm, Marcus R. Makowski, Sarah Keller

**Affiliations:** ^1^Department of Radiology, Charité-Universitaetsmedizin Berlin, Corporate Member of Freie Universitaet Berlin and Humboldt-Universitaet zu Berlin, Charitéplatz 1, Berlin 10117, Germany; ^2^Department of Veterinary Medicine, Institute of Veterinary Pathology, Freie Universität Berlin, Robert-von-Ostertag-Str. 15, Building 12, Berlin 14163, Germany; ^3^Division 1.5 Protein Analysis, Federal Institute for Materials Research and Testing (BAM), Richard-Willstätter-Str. 11, Berlin 12489, Germany; ^4^Department of Chemistry, Humboldt-Universität Zu Berlin, Königin-Luise-Str. 1–3, Berlin 14195, Germany; ^5^Department of Biology,Chemistry and Pharmacy, Institute of Biology, Freie Universität Berlin, Königin-Luise-Str. 1–3, Berlin 14195, Germany; ^6^Institute of Inorganic and Analytical Chemistry, Westfälische Wilhelms-Universität Münster, Corrensstr. 30, Münster 48149, Germany; ^7^King's College London, School of Biomedical Engineering and Imaging Sciences, St Thomas' Hospital Westminster Bridge Road, London SE1 7EH, UK; ^8^BHF Centre of Excellence, King's College London, Denmark Hill Campus, 125 Coldharbour Lane, London SE5 9NU, UK; ^9^Technical University of Munich, Department of Diagnostic and Interventional Radiology, Ismaninger Str. 22, Munich 81675, Germany

## Abstract

**Background:**

Currently, there is no reliable nonsurgical treatment for abdominal aortic aneurysm (AAA). This study, therefore, investigates if doxycycline reduces AAA growth and the number of rupture-related deaths in a murine ApoE−/− model of AAA and whether gadofosveset trisodium-based MRI differs between animals with and without doxycycline treatment.

**Methods:**

Nine ApoE−/− mice were implanted with osmotic minipumps continuously releasing angiotensin II and treated with doxycycline (30 mg/kg/d) in parallel. After four weeks, MRI was performed at 3T with a clinical dose of the albumin-binding probe gadofosveset (0.03 mmol/kg). Results were compared with previously published wild-type control animals and with previously studied ApoE−/− animals without doxycycline treatment. Differences in mortality were also investigated between these groups.

**Results:**

In a previous study, we found that approximately 25% of angiotensin II-infused ApoE−/− mice died, whereas in the present study, only one out of 9 angiotensin II-infused and doxycycline-treated ApoE−/− mice (11.1%) died within 4 weeks. Furthermore, doxycycline-treated ApoE−/− mice showed significantly lower contrast-to-noise (CNR) values (*p*=0.017) in MRI compared to ApoE−/− mice without doxycycline treatment. In vivo measurements of relative signal enhancement (CNR) correlated significantly with ex vivo measurements of albumin staining (*R*^2^ = 0.58). In addition, a strong visual colocalization of albumin-positive areas in the fluorescence albumin staining with gadolinium distribution in LA-ICP-MS was shown. However, no significant difference in aneurysm size was observed after doxycycline treatment.

**Conclusion:**

The present experimental in vivo study suggests that doxycycline treatment may reduce rupture-related deaths in AAA by slowing endothelial damage without reversing aneurysm growth.

## 1. Introduction

Abdominal aortic aneurysms (AAA) are potentially life-threatening diseases with a prevalence ranging from 1.2% to 7.7% in previous screening studies [1–3]. A complication is spontaneous rupture of the aneurysms, as the aortal diameter is exponentially related to aneurysm growth and risk of rupture [4]. More than 95% of aortic aneurysms are located in the infrarenal segments and typically remain asymptomatic over a long period of time.

Extracellular matrix (ECM) proteins such as collagen, elastin, and proteoglycans are important structural elements of the arterial wall [5–7]. Preliminary studies in AAA have shown increased expression of proinflammatory cytokines and matrix metalloproteinases (MMPs), as well as an imbalance between MMPs and their naturally occurring inhibitors [8, 9]. MMPs belong to a class of enzymes that have the primary function of degrading the ECM. Therefore, they play a prominent role in physiological processes such as pregnancy or wound healing [10,11].

Doxycycline is a nonspecific inhibitor of MMPs and functions similarly to endogenous inhibitors [12]. The inhibitory effect is maintained in nonantibiotic chemically modified tetracyclines without antimicrobial activity [13].

The protective effect of doxycycline on the progression of an AAA was previously demonstrated in preliminary studies in a mouse model [14–18]. Due to the rapid metabolism of doxycycline in mice, the weight-adjusted doses in the murine model reached serum levels comparable to those of humans after standard dosing [14]. Depending on the serum concentration, a reduction in aneurysm growth of 33–66% was observed in the mouse model [14]. In a recently published experimental study on the development of aortic aneurysms in Marfan syndrome, the therapeutic efficacy of doxycycline was confirmed [19].

Noninvasive imaging techniques such as magnetic resonance imaging have already been successfully used to evaluate structural components of AAA and potential rupture risks in a mouse model [20–22].

Gadofosveset trisodium is a clinically approved gadolinium-based blood pool MRI contrast agent that is characterized by the high affinity of its ligand (fosveset) to albumin. Due to its albumin-binding capacity, gadofosveset trisodium has an increased relaxivity and extended blood residence time, which is a characteristic of MRI angiography [23]. Furthermore, due to its reversible binding capacity, it enters the vascular wall and interstitium through leaky neovascularization or damaged endothelium [24, 25]. This property of gadofosveset could allow, in addition to the morphological presentation of AAA, quantitative estimates of the extent of endothelial damage, especially with regard to potential therapies such as doxycycline.

To the best of our knowledge, the therapeutic effectiveness of doxycycline on the progression of AAA in the experimental model has not yet been demonstrated by MRI angiography. The aim of this study was therefore to evaluate the effect of doxycycline on AAA progression in a mouse model using gadofosveset trisodium-based MRI angiography in correlation to histopathological findings.

## 2. Materials and Methods

### 2.1. Study Design

All experimental procedures and protocols were performed in accordance with both the guidelines and regulations of the Federation of Laboratory Animal Science Associations (FELASA) and the local Guidelines and Provisions for Implementation of the Animal Welfare Act, which were approved by the Regional Office for Health and Social Affairs Berlin (LAGeSo, registration number: G0,169/15). Nine male homozygous apolipoprotein E-deficient (Apoe−/−) (B6.129P2-ApoEtm1Unc/J) mice were used for this experimental study, being obtained from our research institute of experimental medicine. Results were compared with previously published and studied identical wild-type (C57BL/6J) control animals as well as previously studied ApoE−/− (B6.129P2-ApoEtm1Unc/J) animals after implantation of osmotic minipumps and four-week infusion with angiotensin II [21]. Subcutaneous implantation of osmotic minipumps (Alzet, Model-2004, Durect Corporation, USA), which were set to deliver angiotensin II (AngII) at a rate of 1000 ng/kg/min for up to 4 weeks, was performed to induce the formation of aneurysms. In the present study, doxycycline was administered at 30 mg/kg/d (Sigma-Aldrich) parallel to the continuous administration of angiotensin via the minipumps [26]. Doxycycline-treated animals (*n* = 9) were followed up for 4 weeks before imaging and were imaged before and after contrast at 4 weeks. Imaging was performed before and 30 min after injection of a clinical dose of 0.03 mmol/kg gadofosveset After the imaging session, the tissue was harvested, and histology, immunohistochemistry, and laser-ablation-inductively-coupled plasma-mass spectrometry (LA-ICP-MS) were performed.

### 2.2. In Vivo Magnetic Resonance Experiments

Imaging was performed in supine position with a clinical 3T Siemens MRI scanner (Biograph mMR, Siemens-Healthcare-Solutions, Germany) using a 4 channel receive coil array for small animal imaging (Mouse Heart Array, Rapid Biomedical GmbH, Germany). An overview of the MR scan parameters can be obtained from Table 1. To avoid cooling and reduced body temperature during MRI, the body temperature (37°C) of the mice was monitored using a MR-compatible heating system (Model 1025, SA Instruments Inc, NY). For venous access, a small diameter tube with a 30G cannula attached to it was used, which allowed the injection of contrast medium into the tail vein.

### 2.3. Magnetic Resonance Imaging Analysis

MR image analysis was performed using Visage (version 7.1, Visage Imaging, San Diego, CA). 2D regions of interest (ROIs) were drawn around the enhancing vessel or aneurysmal wall in delayed-enhancement MR images, whereby anatomical colocalization with high-resolution MR angiography images was performed. In the delayed-enhancement sequences (gadofosveset), the signal from the blood was suppressed. Contrast-to-noise ratios (CNR) were calculated as follows, where noise was defined as the standard deviation of the background ROI in the air anterior to the aorta:

(1) $$\begin{array}{c} \text{CNR}=\frac{\text{combined vessel wall and aneurysmal signal intensity}-\text{blood signal instensity}}{\text{standard deviation of the image noise}}. \end{array}$$

### 2.4. Anesthesia and Tissue Harvesting

Anesthesia was performed by intraperitoneal injection of a combination of medetomidine (500 *μ*g/kg), midazolam (5 mg/kg), and fentanyl (50 *μ*g/kg). After the implantation of the minipumps, a reversal agent consisting of atipamezole (2.5 mg/kg), flumazenil (500 *μ*g/kg), and naloxone (1200 *μ*g/kg) was administered to terminate the anesthesia [27]. Euthanasia was performed under anesthesia by dislocation of the cervical spine. This was followed by the opening of the situs and terminal exsanguination by perfusion through the left ventricle with physiological saline solution for 10 minutes.

### 2.5. Histology and Immunohistochemistry

After euthanasia and opening of the situs, the aortic tissue was removed and immediately embedded in a tissue freezing medium (OCT compound) for subsequent cryosectioning. Abdominal aortic tissue was cut into 10 *μ*m cryosections at −20°C and then mounted on SuperFrost Plus adhesion slides (Thermo Scientific). The nine animals were sacrificed after 4 weeks of angiotensin II infusion and doxycycline treatment. Transverse sections (7 *μ*m) were obtained at multiple sites along the aorta in 200 *μ*m intervals. Cryosections were stained with hematoxylin-eosin (H&E) for overall imaging of tissue components and with Miller's Elastica-van-Gieson (EvG) for imaging of elastic fibers. In addition, immunofluorescence staining was performed with polyclonal primary anti-mouse serum antibodies (Goat polyclonal Mouse Serum Albumin, ab19194, Abcam, Australia, 1:100), which were incubated with AlexaFluor 568-labeled polyclonal secondary antibodies (Goat anti-Rabbit IgG, Thermo Fisher Scientific, Germany, 1:500). Fluorescence-positive regions were assessed on digital images using ImageJ (version 1.51, National Institutes of Health). The fluorescence-positive region was then segmented and expressed as a percentage of the total adventitious area.

### 2.6. Laser Ablation-Inductively Coupled Plasma-Mass Spectrometry

For elemental bioimaging, the LA-ICP-MS was performed with a LSX-213-G2+ laser system (CETAC Technologies, USA) that was equipped with a two-volume HelEx II cell. The latter was connected to an ICP-MS spectrometer (ICPMS-2030, Shimadzu, Japan) via Tygon tubing. Quantification and visualization were performed with software developed in-house (Robin Schmid, WWU-Münster, Germany). The histological samples were ablated via line-by-line scan and a spot size of 15 *μ*m, scanning speed of 45 *μ*m/s, and 800 mL/min Helium as carrier gas. The subsequent analysis was performed in collision gas mode with helium as collision gas and 100 ms integration time for the isotopes to be analyzed (^31^P, ^64^Zn, ^160^Gd, and ^158^Gd). For the quantification of gadolinium, matrix-matched gelatin-based standards were used. Nine gelatin standards (10% w/w), including a blank, were spiked with different gadolinium concentrations (1 to 5.000 *μ*g/g). The limit of detection and the limit of quantification (3*σ*- and 10*σ*-criteria) were 8 ng/g and 28 ng/g Gd, respectively. There was a good linear correlation for the averaged intensities of the scanned lines with a regression coefficient of *R*^2^ = 0.9999 within this concentration range.

### 2.7. Statistical Analysis

Statistical analysis was performed with the statistical software “*R*” (version 4.0.3, *R* Development-Core-Team, 2015). Bar charts with standard deviations were used for the visual illustration of CNR values. A *p* value ≤0.05 was considered statistically significant.

## 3. Results

**Figure 1:**
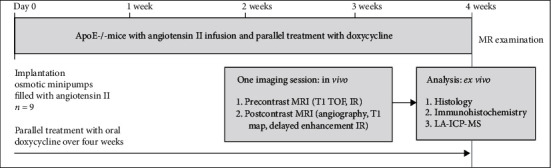
Overview of the experimental in vivo study design. The present experimental study included 9 ApoE/-mice, who were implanted with osmotic minipumps with subsequent release of angiotensin II and were treated in parallel with doxycycline for four weeks. After 4 weeks, mice received an MR imaging examination, which included precontrast (unenhanced) imaging (T1 TOF and IR) and postcontrast imaging (angiography, T1 map, and delayed enhancement IR). The mice were then sacrificed and histology, immunohistochemistry, and LA-ICP-MS were performed. LA-ICP-MS: laser ablation-inductively coupled plasma-mass spectrometry.

**Figure 2:**
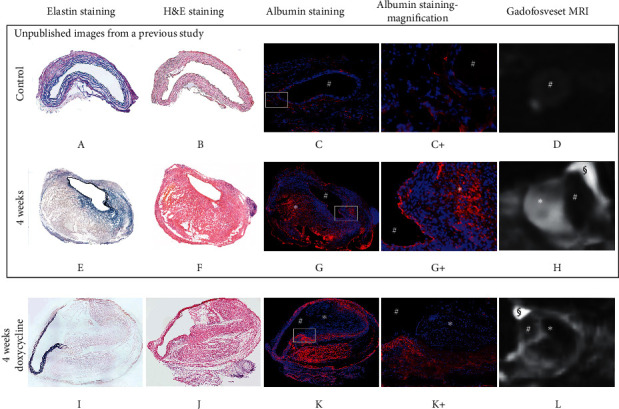
Example of in vivo molecular MRI with an albumin-binding probe for the visualization of controls and aortic aneurysms in an animal model. Images from control and 4-week animals (A–J) are unpublished images from a previous study [21]. # indicates the vessel lumen, *∗* indicates the thrombus area (both nonenhancing organized thrombus and not fully organized thrombus), and § indicates an imaging artifact incomplete. A corresponds to control aortic tissue stained with elastin. B shows aortic tissue from the same probe stained with hematoxylin and eosin (H&E). C and the corresponding magnification image C+ correspond to albumin-specific staining with small immunopositive fluorescent areas of albumin. D is delayed-enhancement imaging following the administration of the albumin-binding probe gadofosveset with minimal signal enhancement. E demonstrates an aortic aneurysm after four weeks of angiotensin II infusion using elastin staining. F is a section from the same probe, stained with H&E. G and the magnification image G+ correspond to albumin-specific staining with confluent immunopositive fluorescent areas of albumin, indicating an extracellular accumulation of albumin. H is delayed-enhancement imaging following the administration of the albumin-binding probe gadofosveset with strong signal enhancement of the AAA vessel wall/thrombus area. I corresponds to an aortic aneurysm after four weeks of angiotensin II infusion and parallel treatment with doxycycline, stained with elastin. J demonstrates an aortic aneurysm with H&E staining. K and K+ correspond to the albumin-specific staining with confluent immunopositive fluorescent areas of albumin, indicating an extracellular accumulation of albumin. L is delayed-enhancement imaging following the administration of the albumin-binding probe gadofosveset with intermediate signal enhancement.

Figure 1 gives an overview of the in vivo experimental study design. The animals were 12 ± 1 weeks old and drug-naïve with a weight between 28 g and 32 g. Sham-operated mice with a continuous saline infusion for 4 weeks served as controls and showed no signs of AAA development (controls reported in a previous publication [21]). After intravenous contrast administration of gadofosveset, the aneurysmal vessel wall showed an intermediate to strong enhancement, while the blood signal was nulled. The increased enhancement from the albumin-binding probe gadofosveset corresponded to the ex vivo immunofluorescent albumin staining (also refer to Figure 2).

### 3.1. Survival and Response to Therapy

In a previous study, we found that approximately 25% of ApoE−/− mice died before MR imaging after implantation of osmotic minipumps and infusion of angiotensin, whereas none of the wild-type C57BL/6J animals died [21]. By comparison, only one out of 9 ApoE−/− mice (11.1%) died within 4 weeks before imaging after parallel treatment with doxycycline. The cause of death was determined by postmortem autopsy and resulted from aneurysm rupture. These results indicate that doxycycline may reduce the risk of rupture and thus mortality in murine aortic aneurysms.

**Figure 3:**
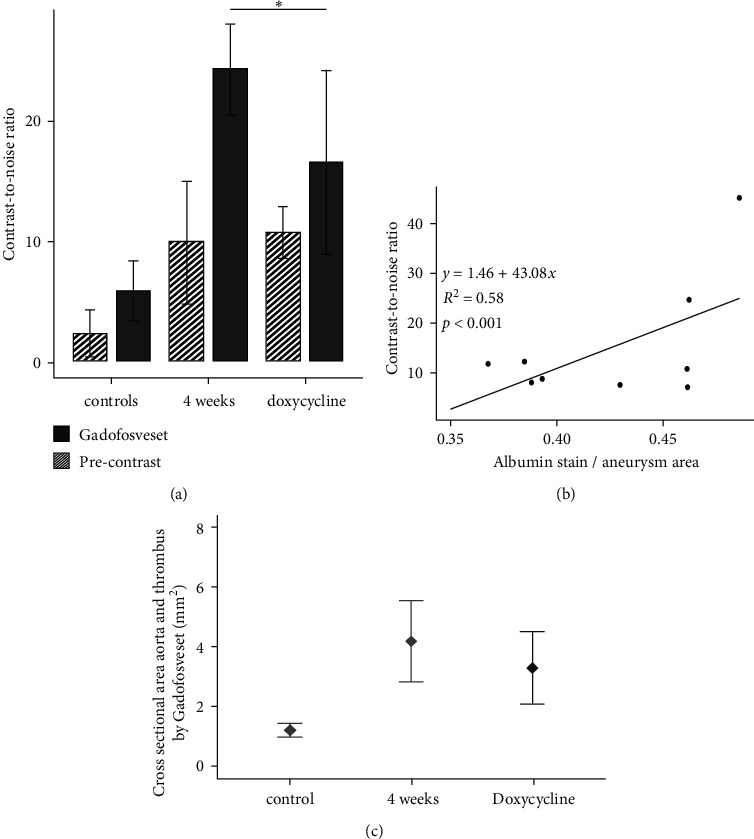
Graphic visualization of MRI measurements. (a) Contrast-to-noise ratios measured in control animals and in ApoE−/− mice after 4 weeks of angiotensin II infusion and after 4 weeks of angiotensin II infusion with parallel treatment of doxycycline. Compared to controls, postcontrast gadofosveset-enhanced contrast-to-noise ratios show increased signal enhancement (controls and 4-week group previously published in Scientific Reports, 2020 [19]). What becomes apparent in the present analysis is that doxycycline-treated animals show lower contrast-to-noise ratios compared to animals after 4 weeks of angiotensin II infusion (*p*=0.017). (b) Furthermore, ex vivo relative fluorescence albumin-stained areas also showed a significant (moderate) correlation with in vivo measured contrast-to-noise ratios (*R*^2^ = 0.58). (c) In vivo MRI combining cross-sectional areas with associated thrombus areas also show a significant increase in size in four-week-old aneurysms compared to controls. Doxycycline-treated aneurysms appear slightly smaller; however, this difference was not significant (*p*=0.275).

However, regarding microscopic aneurysm and thrombus size, there was no significant difference between angiotensin II-treated and angiotensin II and additionally doxycycline-treated animals (*p*=0.069). On the other hand, in vivo MR measurements showed that mice receiving a pharmacological intervention with doxycycline treatment in addition to angiotensin II infusion showed significantly lower CNR values (*p*=0.017) compared to ApoE−/− mice without doxycycline treatment after 4 weeks of angiotensin II infusion (ApoE−/− mice without doxycycline treatment previously reported and published [21]). In vivo relative signal enhancement measurements (CNR) significantly correlated to ex vivo albumin stain area measurements (Figure 3(b)). In addition, the size of albumin-positive areas in ex vivo immunohistochemistry was smaller in doxycycline-treated animals compared to ApoE−/− mice without doxycycline treatment (39 ± 4% vs. 49% ± 7%). By comparison, it was previously shown that there are only minor immunopositive fluorescent areas of albumin in the aorta of wild-type animals, especially in the adventitia [21] (also refer to Figure 3).

### 3.2. LA-ICP-MS

**Figure 4:**
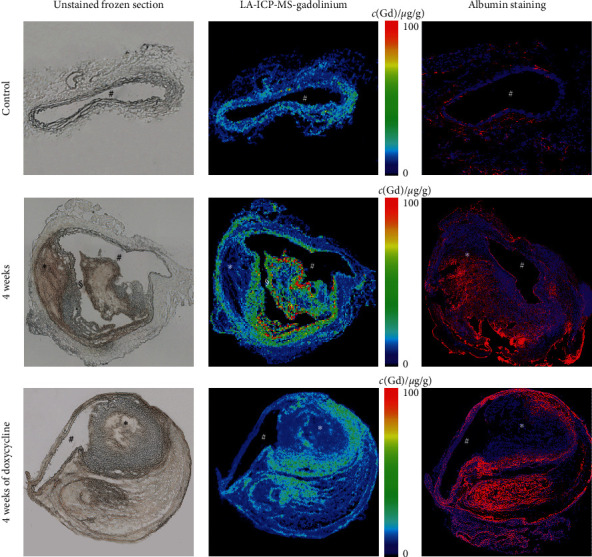
Ex vivo results from LA-ICP-MS. A–I are unpublished images from a previous study [21]. # indicates the vessel lumen, *∗* indicates the thrombus area (both nonenhancing organized thrombus and not fully organized thrombus), and § indicates an area where the cryosection tore during preparation. A, D, and G correspond to the unstained frozen sections. B, E, and H show the spatial distribution of gadolinium (from gadofosveset) in aortic aneurysms assessed by LA-ICP-MS. Brighter colors in the color bars on the right (red and yellow) correspond to higher gadolinium concentrations with the highest measured gadolinium concentration being 120 *μ*g/g (red color). C, F, and I correspond to albumin-specific staining with minor C and confluent (F–I) immunopositive fluorescent areas of albumin.

The gadolinium distribution was visualized by LA-ICP-MS. AAA sections of ApoE−/− mice with 4 weeks of AngII-infusion and doxycycline treatment showed a strong colocalization of targeted gadolinium with immunopositive fluorescent areas of albumin (Figure 4). Confirming the observations for immunofluorescence albumin-staining, an increase in gadolinium concentration could be observed in ApoE−/− mice with 4 weeks of angiotensin II infusion compared to control animals. Besides, there was a strong visual colocalization of albumin-positive areas in the fluorescence albumin stain with gadolinium distribution in LA-ICP-MS (Figure 4). Doxycycline-treated animals also showed a higher gadolinium concentration than control animals, but it appeared reduced compared to animals with 4 weeks of angiotensin II infusion (Figure 4). Accumulation of extraluminal albumin may result from a damaged endothelium with increased endothelial gap junction in abdominal aneurysms, whereby the progression of this damage might be slowed down by doxycycline treatment.

## 4. Discussion

The main findings of this study are that doxycycline may have a positive effect on vessel wall stability in angiotensin-induced AAA in a mouse model with a subsequently reduced mortality.

Compared to other extracellular contrast agents, such as gadolinium-diethylenetriamine penta-acetic acid (DTPA), gadofosveset reversibly binds to serum albumin at 37° body temperature, thus enabling a 3–5 times higher relaxivity and prolonged intravascular enhancement at 3 Tesla [28]. The high intravascular enhancement and the long intravascular residence time can be used to achieve higher signal-to-noise ratios with otherwise constant parameters or increased spatial resolution with a constant signal-to-noise ratio [28]. In patients undergoing endovascular repair of AAA, gadofosveset showed higher detection rates of endoleakage as a sign of endothelial instability [29].

Doxycycline as a known inhibitor of matrix-metalloproteinases [14] has been previously shown to reduce the progression of AAA in murine models [14–18]; however, to date, no MRI-based angiographic studies have been performed to reflect this.

In this study, the ApoE−/− mouse, a common model of abdominal aortic aneurysm, was used. Doxycycline at a clinical dose of 30 mg/kg/KG/d was administered in parallel with implantation of the aneurysm-inducing angiotensin-II minipumps. As an albumin-binding extracellular contrast agent, gadofosveset showed a strong signal in the disrupted areas of the aneurysm wall in all animals, which could be quantified by CNR. The protective effect in the doxycycline group was shown, as expected, by a significantly reduced CNR in the gadofosveset-enhanced MR angiography compared to the untreated control group from the previous study [21]. This could be interpreted as a sign of reduced endothelial damage in this group and thus reduced leakage of albumin and consecutively gadofosveset into the aneurysm wall. These observations were supported by immunohistochemical analysis, which confirmed the decreased areas of albumin in the aneurysm wall of the doxycycline group ex vivo.

Consistent with these results, we observed improved survival rates in doxycycline-treated mice compared with untreated ApoE−/− animals after 4 weeks of Ang II [21].

An interesting finding was that doxycycline-treated and untreated ApoE−/− mice did not show any significant difference in aneurysm size. This stands in contrast to prior studies in animal models that reported inhibition of aortic growth by up to two-thirds [14, 18, 30, 31]. In the clinical setting, discrepant results have been reported for oral doxycycline therapy in AAA, with two studies observing a significant reduction in aortic aneurysm diameters 6–12 months after 3 months of therapy [32, 33], whereas a randomized clinical trial reported a significant increase in aneurysm diameters in the doxycycline group 18 months after initiation of therapy [34]. A recent randomized clinical trial including 261 patients with AAA found that, among patients with small AAA, doxycycline did not significantly reduce AAA growth at two years compared with placebo [33]. This was in line with the results from Meijer et al., who also reported no effect of doxycycline on AAA growth or need/time to AAA repair [34]. We, therefore, hypothesize that the protective effect of doxycycline on the endothelial wall does not necessarily lead to a slowing of aneurysm growth, but possibly to increased wall stability due to a decreased degradation of important structural proteins, which in turn could reduce the risk of a fatal outcome of aneurysm rupture.

The present study has some limitations. First, the study cohort with 9 animals in the doxycycline group was relatively small. One reason for this is the animal welfare approach to still achieve sufficient statistical power with the smallest possible number of animals. One of the most important limitations is the difference between murine and human abdominal aortic aneurysms. While human abdominal aortic aneurysms are localized in the infrarenal aorta, AAA in mice develops in the suprarenal portion of the aorta, possibly reflecting regional differences in the composition of the extracellular matrix, which in turn could lead to altered mechanical properties [35–37]. However, the structural remodeling of the adventitia induced by angiotensin II infusion in the ApoE−/− mouse model has similar characteristics to those in human disease [38]. In addition to the structural changes of adventitia, the AAA of the ApoE−/− mouse model also shows other features consistent with human disease, such as mononuclear inflammatory cell infiltration, neovascularization, and elastic laminar destruction [39–41]. Apart from the inhibitory effect on MMPs, doxycycline has also been shown to have anti-inflammatory and immunomodulatory effects [13]. In another mouse model for AAA, doxycycline effectively suppressed neutrophils, macrophages, and lymphocytes in the aortic wall, with macrophages being particularly associated with the progression of aortic aneurysms [42]. The extent to which doxycycline also has a positive effect on associated inflammatory activity in this model would be an interesting question for follow-up studies.

## 5. Conclusions

The results of this MR angiography study show that the administration of doxycycline in the murine model might have a protective effect on the rupture risk of AAA, possibly through the stabilizing effect on the aneurysm wall and the associated reduction in endothelial leakage. Gadofosveset as an albumin-binding extracellular contrast agent is suitable for the specific visualization of this endothelial leakage and thus creates a direct image-morphological comparison between therapy and control group.

## Figures and Tables

**Table 1:** Tabulated imaging parameters.

Sequence	2D TOF	2D TI sequence	3D IR FLASH sequence
Scan plane	Axial	Axial	Axial
Voxel size (mm)	0.2 × 0.2 x 0.5	0.6 × 0.6 × 3	0.1 × 0.1 × 0.3
Number of slices	40	31	56
TR/TE (ms)	35/4.44	44.91/2.09	1019.72/7.02
FoV (mm)	200	340	57
Flip angle (°)	90	35	30
Matrix	960	576	384
Bandwidth (Hz/Px)	124	579	130
TR between IR pulses		1000	1000

## Data Availability

The data used to support the findings of this study are included within the article and available from the corresponding author upon request.
